# Response—Liminality and the Mirage of Settlement

**DOI:** 10.1007/s11673-021-10160-w

**Published:** 2022-04-01

**Authors:** Claire Hooker, Ian Kerridge

**Affiliations:** Sydney Health Ethics, Sydney School of Public Health, Faculty of Medicine and Health, University of Sydney, A27 Room 107, Sydney, NSW 2006 Australia

**Keywords:** Bioethics, Liminality, Illness Experience, Existential philosophy

## Abstract

Little and colleagues’ (1998) paper describing a key aspect of cancer patients’ experience, that of “liminality,” is remarkable for giving articulation to a very common and yet mostly overlooked aspect of patient experience. Little et. al. offered a formulation of liminality that deliberately set aside the concept’s more common use in analysing social rituals, in order to grasp at the interior experience that arises when failing bodily function and awareness of mortality are forced into someone’s consciousness, as occurs with a diagnosis of cancer. We set out the reasons as to why this analysis was so significant in 1998—but we also consider how the “liminality” described by Little and colleagues was (as they suggested) a feature of modernity, founded on what we term “the mirage of settlement.” We argue that this mirage is impossible to sustain in 2022 amid the many forms of un-settling that have characterized late modernity, including climate change and COVID-19. We argue that many people in developed nations now experience liminality as a result of the being forced into the consciousness of living in a continued state of coloniality. We thus rejoin the social aspects of liminality to the interior, Existential form described by Little et. al.

One of the most striking features of the paper published twenty-three years ago by Miles Little with colleagues Chris Jordens, Kathleen Montgomery, Kim Paul, and Bertil Philipson—“Liminality: A major category of the experience of cancer illness” (Little et al. 1998)—is, in retrospect, the obviousness of the concept of liminality in articulating a key element of the experience of serious illness. But though obviousness might make a paper superficial or trite, this one described a phenomenon in ways that make the intangible, tangible; the invisible, visible; the complex, simple; and the unspoken, heard.

The second striking feature of Little and colleagues’ work is that the experience of liminality that they describe in their patient population is not a rare experience but one that forms (at least a part of) the identity and meaning-making of many people who face the challenge of serious illness. Indeed the commonness of the experience of liminality is borne out by the fact that, following Little, it has been described in a range of different settings and contexts, including in people with terminal illness, advanced prostate cancer, HIV/AIDS, chronic pain, kidney failure, spinal injury, heart disease, chronic pulmonary disease, and so forth (see e.g. (Berger and Kroesen 2016; Blows, et al. 2012; Brown 2018; Dawson, Adams, and Fenlon 2019; Ganteau and Onega Jaen 2014; Jackson 2005; Kelly 2008; Philip et al. 2014; Willig and Wirth 2018; Willig 2019). Remarkably, despite this, this feature experience is not commonly referred to or described in patient or physician accounts of illness. At least one news organization felt the need to explicitly reintroduce the idea in relation to COVID-19, telling its readers that the experience of the “shifting sands” of the pandemic “has a name—liminality” (Wayland 2021; see more below).

This matters because, as Little and colleagues recognized at the time, the measures used by clinicians and health agencies to identify health outcomes, including “quality of life” measures, describe very, very little of actual illness experience. Indeed one of Miles Little’s most significant contributions was to insist on, and actively advocate for, shifts towards patient-centred care and for attention to patients’ values and experience in both clinical care and the evaluation of medicines.

While there has been some progress toward these goals, the “absence” of the patient from medical care remains as relevant an issue today as when the paper was published, because the tools and measures that define and constrain healthcare not infrequently still fail to grasp much of the qualia of patient experience. Indeed, vast increases in information surveillance tools can ironically create *greater* gaps between what the tools measure (and why) and what either patients or their clinicians actually value.

The third striking feature of this paper is its methodology. In seeking an answer to the question “How can we best capture the subjective and embodied experience of suffering and illness, in ways that enrich research, education and practice?” Little and colleagues drew insights from both a careful examination of the illness narratives of patients with cancer and from theoretical and empirical literature. Their analysis arose from the empirical richness of narrative accounts of illness such as those of Frank and Kleinman (Frank 1995; Kleinman 1988). The authors collected a heterogenous corpus of texts, including published accounts and extensive clinical experience in addition to a small number of interviews (motley inclusion criteria that might be challenged today). To analyse this data, they sought theoretical, conceptual, and methodological insights from continental philosophers and linguists including Kierkegaard, Heidegger, Satre, de Beauvoir, Merleau-Ponty, Foucault, and Derrida, thinkers relatively under-represented in contemporary bioethics (Hanna 1998). From these sources Little et. al. constructed a kind of genealogy of illness experience in the traditions and history of Existentialist philosophy, exploring its construction through the discourse in which it was applied, that is, in illness narratives.

“Liminality” is a concept founded in metaphor: the metaphor of the threshold, the point of passing over from one state or situation to another (for a comprehensive discussion of the concept and its linguistic representation from the ancient world on, see Horváth, et al. 2015). The metaphor/concept has enjoyed considerable uptake, particularly in European anthropology and cultural studies. Little and colleagues refer to the famous early twentieth century anthropologist Van Gennep’s studies of the functions of ritual in non-White societies (Gennep 1977) and to late twentieth century anthropologist and performance theorist Victor Turner’s adaptation of Van Gennep to examine “public” liminality in theatre and performance, which included the capacity to “invert” social roles or status (Turner 1980). Van Gennep’s and Turner’s social functionalist conceptualization of liminality identified *phases* of liminality, which eventually result in a new state or a resolution. That is: resolution, the completed crossing of the threshold, is central to both models. These phased conceptions of liminality remain useful in understanding health contexts, as we know from our own work (Dalton, et al. 2020; Kowalski, Hooker, and Barratt 2019). While there has been a resurgence of interest in the concept of liminality in the past ten years, this literature is dominated by social and political transitions (Horvath, Bena, and Davison 2018; Horváth et al. 2015; Lamond and Moss 2020; Szakolczai 2017; Thomassen 2016), rather than the inchoate qualia of personal, even private, experience.

Importantly, having settled upon the salience of liminality to making sense of the subjective experience of cancer, Little and colleagues do not then simply apply this concept but extend and rework it. The liminality described by Little et. al. is not defined as a series of stages or phases of transition in the manner of Van Gennep or of reaching resolution, in the manner of Turner. Instead, their analysis was attuned to capturing the essence of liminality, which is the in-between, the hovering on the threshold. They explicitly conceived liminality as both interior and as proces*s*, acknowledging that it could be a long-term or even lifelong (Blows, et al. 2012) existential state, into which people entered as result of a diagnosis of serious illness. The phenomena they describe is messy, unstructured, persistent, discordant, contradictory, and “lived” rather than ritualized and, most significantly, imagined as a “normal” process of illness experience rather than as psychological pathology. Liminality describes an experiential space that is neither singular or unidirectional but has the potential for distress and joy, for collapse and growth, for resolution or persistence, and for disconnection and connection (Kelly 2008; Willig 2019).

The key feature of liminality as Little and his colleagues recognized it, was the discombobulating disruption of identity and action caused by awareness of bodily function as those functions falter or fail, along with the confronting and vertiginous awareness of mortality. Perhaps unsurprisingly, this, along with exclusion from normed social categories and identities, meant that the concept of liminality was used to describe the experience of disability prior to Little et. al.’s research (Hahn 1988; Murphy, et al. 1988). Indeed, Little et. al.’s paper might have been stronger had it engaged with this scholarship—but it is worth noting that Little’s prioritizing of patient experience in medical research was part of, and contributed to, understanding the value of knowledge that arises from lived experience in health and medicine (and beyond), which was also powerfully fuelled at that time by Disabilities scholars and activists. This orientation to “knowledge-holders” as well as researchers, is only now reaching its full flowering (e.g. (Bellingham, et al. 2021)). Since the liminalities paper’s publication, scholars, activists, and artists with disability have increasingly, and creatively, contested previous understandings of liminality. Indeed, this scholarship now raises the question of whether the notion of liminality may actually misrepresent or misunderstand disability or entrench ableism (Dorwart 2017; Hughes 2005; Kuppers 2014).

## Liminality and Un-Settling

If we presume that liminality is universally experienced by patients with a serious and potentially life limiting illness, the state must be a very common one. While this seems self-evidently true, we suggest that the concept’s explanatory power and relevance may extend far beyond the context of life limiting illness. Arguably, indeed, *any* illness, or *any* circumstance that suddenly forces the body out of what Little and his colleagues termed “transparency” (i.e., the capacity to be oblivious to it because of its seamless functioning), induces at least a temporary state of liminality. Hence, liminal states are experienced by us all. They must be, because, as Disabilities scholars remind us e.g. (Mackenzie, Rogers, and Dodds 2013), the transparent body is in fact the exception, rather than the norm it seems to be.

This bears a little thinking. One could imagine that if the notion of liminality is understood too broadly, such that even a blocked nose induces a state of liminality—this may weaken the concept. In response, we suggest that liminality occurs when the body is not only forced on awareness but when this awareness also disturbs or threatens a fundamental aspect of identity. Thus, even a blocked nose can do that if, for example, it prevents the sufferer from inhabiting their professional identity temporarily, as may happen is they work as a chef, cheesemaker, or sommelier. Living with a permanently threatened social identity because one’s body does not fit powerful social norms may also place one in a state of liminality.

Following this train of thought, the concept of liminality draws our attention to what we might refer to as the *mirage of settlement.* Our choice of the word “settlement” here is informed by recent de-colonizing scholarship which frames analysis around colonized and “settler” societies, understanding the latter as interlinked systems of power and representation In their paper, Little and colleagues noted that “[t]he non-liminal existence is a creation and ideal of modernity, and postmodernity’s deconstructions seem to have left the non-liminal ideal untouched.” This passing phrase is, perhaps, the most philosophically profound and far-reaching observation the authors make, and remains one of the most resonant. Let us consider the latter clause first. Even in 1998, we suggest, “postmodernity’s deconstructions” *had* begun to unsettle the non-liminal ideal. This project was undertaken explicitly by feminist theorists, who anatomized how modernity’s illusions of normality, autonomy, and stability were constructed through discourse (Irigaray 1980), explored the leakiness and non-conformity of female bodies, a contrast to illusory masculine norms (Shildrick 2015), showed the ideal had to constantly repress the unspeakable *abject* (Kristeva 1982) and, above all, recognized the non-liminal ideal—a cognitive, rational, active, autonomous subject—for the masculine fantasy that it was (Phillips and Barrett 1992).

The version of liminality that occurs when people grasp that what had seemed both normal and stable, that is, *settled*, is in fact not only unrepresentative and particular, but also temporary, dynamic and transient—that “entities” are better understood as complex dynamic systems, or networks, or in-process processes—is, we suggest, a distinctive characteristic of contemporary human circumstances. Epistemically, this has indeed been the project of post-modern and non-representational scholarship e.g. (Andrews, 2018). Re-conceptualizing the world as in a state of *flux*—un-settled, polymorphous, contested, and dynamic—has occurred at the level of our bodies: for example, recent medical science telling us that we are in a constant state of turmoil and wrongness, with our constant internal cell replication generating thousands of cancer-causing mutations daily (Tomasetti, Li, and Vogelstein 2017). Or social science illuminating for us that sex is no longer assumed to be stably dimorphic but acknowledged to be variable, flexible, impermanent, and determined (Schroedinger-like) by our own examination of it (e.g. Carpenter 2020).

This reconceptualization has also occurred at the macro level of global politics (see (Horváth, et al. 2015). Flux, and its concomitant desiderata, agility, are now inescapable features of contemporary life in developed nations, in a way they were not in 1998. People working in gig economies and characterized by (or recommended to build) “portfolio careers” (Platman 2004)—the relentless entrepreneurs of the self (Giddens and Pierson 2013)—are increasingly un-settled. Their living circumstances are precarious. Their identities, so closely linked to professional activity and attainment (the identity politics of late capitalism), are both as brittle as their careers and constantly performed as an alternative substitute stability in the face of fragile professional trajectories (Vallas and Christin 2018).

These thoughts are borne in upon us as we write; COVID-19 is the dominant event of this moment, characterized by significant and multiple un-settling, affecting both selves and societies (see e.g. Awdish 2020). At this moment liminality is a global condition, perhaps experienced by all humans, and driven above all by the consequences of extractive capitalism: COVID-19 (Wayland 2021), climate change, and the worsening political and social instabilities around the world (similar thoughts have prompted recent use of the concept of liminality in contemporary political science, e.g. Thomassen 2016).

In this regard, if Little et. al.’s cancer survivors entered the state of liminality as a result of being forced (due to pain and lack of function) into consciousness of the hitherto transparent body, then many people in the developed world have entered liminality as a result of being forced into painful consciousness of hitherto transparent modernity. We do not live in perpetually young, smoothly functional bodies, and similarly we do not live in perpetually smooth material, progressive ease. Many people have now grasped the fact that instead, we live in a state of coloniality (Moraña, Dussel, and Jáuregui 2008), i.e., in the living legacy of colonialism, characterized by the ongoing and intersecting oppressive states of racism, hetero-patriarchy, ableism, and “place-ism” (Bateman and Pilkington 2011; Meissner and Whyte 2018). The devastating 2019 rainforest conflagrations that preceded COVID-19 in the Amazon and in Australia, and the forest fires that consumed two pandemic summers across the Northern hemisphere, brought mortality into the consciousness of the millions of humans who witnessed their extinguishing of billions of non-human lives. Millions of people have suddenly grasped that “we” the formerly settled modern selves, will soon join non-human animals, First and Coloured peoples, by having our beloved landscapes and ways of life and being, despoiled.

Little and colleagues rightly described how recognizing and exploring the liminal nature of cancer survival provided a distinct and important difference from the way the concept was used by Van Gennep and Turner to explore social processes. But in the face of climate change, ecological destruction and pandemic, we no longer have what might now be termed “the privilege” of *not* connecting the liminality arising from awareness of the body and of mortality, with that arising from awareness of “the strategies around which we have constructed our lives” on the other. “Liminality” might particularly describe the current conditions of White scholarship—Existential philosophers and their scions—and its institutions. The mostly White academics in the Universities of the English-speaking developed world are only just starting to come to grips intellectually with the coloniality of dominant knowledge systems and their institutional foundations, as set out by their Black, First Nations, Disabled, and other subaltern colleagues. At the same time, they are forced to reckon with the same forces in their own lives. The pandemic has fairly brutally revealed the extractive economic base (Petras and Veltmeyer 2014) beneath the superstructure of Western scholarship and the intensified precarity of intellectual laborers (Bone 2020).

## Living with Liminality

But this does not mean that the interior liminality described by Little et. al. is either a now-vanished historical phenomenon or that its private, interior character is unimportant or illusory. Although Existentialist philosophers held the view that “liminality is the mode of life in which we must live” (Little, et al. 1998)—a fact forced upon us by having to live in landscapes under cataclysmic, climate-change-induced transition—it is impossible to live continually in this awareness.

Existentialism did not, contrary to the ways in which it is frequently (mis)represented, require submission to the notion of impermanence and inevitable destruction but pointed to the unavoidable necessity for humans to recognize liminality in all its manifestations and *decide* who they should be. Intriguingly, they saw this decisioning not only as an ethical and epistemological process but also as a public and aesthetic one—actioned and expressed, first and foremost, in art.

… the ways in which a human consciousness “intends” the world … is intrinsically dependent on the values the person has set for herself … The freedom required by the world is first of all that of the artist. Every artwork reveals a fundamental, existential attitude towards the world and is the expression of an existential choice. (Duranty 2019, ¶3 "4. Art as expression of human freedom")

In light of this, following the lead of Disabled or First Nations or climate artist-activist/scholars (Kuppers 2020), not to mention the many creative arts programmes now integrated with cancer care (Ennis, Kirshbaum, and Waheed 2018), we might suggest that many people are finding Un-settling to be a process that is joyous, fierce, energizing, or revelatory (Rogaly and Schling 2021; Roy 2020). Though *liminality is the mode of life in which we must live* and though dread, anguish, and anxiety accompanies this knowledge—it is also creative, hopeful, productive, and powerful.
